# Supplementary material for: Are carbon emissions trading and green financial instruments synergistic? -Comprehensive quantitative research based on content analysis
Source: PLoS One. 2024 Mar 7;19(3):e0298601. doi: 10.1371/journal.pone.0298601 (PMC10919601; doi:10.1371/journal.pone.0298601)
Supplement: S1 Data — (ZIP) [file pone.0298601.s001.zip › Data/地级市Z_F工作报告.zip!/╕ú╜¿/╕ú╓▌╩╨/└┤╫╘╣1⁄2╓┌║┼í╛┬φ┐╦╩2╛▌═°í┐_2016.docx]

——2016年2月16日在福州市第十四届人民代表大会第五次会议上

　　福州市人民政府市长杨益民

各位代表：

　　现在，我代表福州市人民政府，向大会报告政府工作，请予审议，并请市政协委员和其他列席人员提出意见。

　　一、2015年工作暨“十二五”发展回顾

　　2015年，在省委、省政府和市委的正确领导下，我市各级政府全面贯彻党的十八大和十八届三中、四中、五中全会精神，深入贯彻习近平总书记系列重要讲话精神和对福建、福州工作的重要指示，认真落实中央支持福建加快发展的重大政策措施和市委十届十次、十一次全会精神，全力以赴稳增长、调结构、促改革、惠民生，各项工作取得了新进展新成效。据统计，全市生产总值5618.1亿元，增长9.6%；一般公共预算总收入848.04亿元，增长8.7%，地方一般公共预算收入560.46亿元，增长9.7%；固定资产投资4853.61亿元，增长10.6%；外贸出口1312.3亿元，增长0.6%；实际利用外商直接投资16.79亿美元，增长8.5%；社会消费品零售总额3488.74亿元，增长14%；居民消费价格总水平上涨1.7%；城镇居民人均可支配收入34982元，增长7.8%；农村居民人均可支配收入15203元，增长8.5%；城镇登记失业率2.44%；人口自然增长率7.4‰；省下达的节能减排任务全面完成。以上指标多数居于全省前列，但有6项指标没有达到我市预期目标。一年来的主要工作和成效是：

　　（一）积极应对困难，经济保持平稳健康发展。深入开展“三比一看”、“四个万家”等活动，全市各级党员干部下基层约45.8万人次，协调解决问题约5.9万个。及时出台一系列稳增长政策措施，巩固经济基本面。扩大有效投资，595项市级重点项目完成投资2367.17亿元，为年度计划的115.2%；981项行动计划重大项目完成投资2555.05亿元，为年度计划的114.7%。金融机构各项贷款余额增长13.8%，企业直接融资1161.83亿元，有力支持了实体经济发展。

　　（二）转变发展方式，产业转型升级步伐加快。坚持抓龙头、铸链条、建集群，60项重点工业项目竣工投产，26项重点工业项目动工建设，京东方8.5代面板等一批重大产业项目落地，规模以上工业增加值1927.9亿元，增长8.8%。全市技改投资790亿元，增长4.6%。福州高新区、临空经济区、闽台（福州）蓝色经济产业园、台商投资区、江阴工业集中区等园区建设提速，福州经济技术开发区获评国家级物联网园区。加快发展现代物流等14个服务业重点产业，跨境电商公共服务平台投入运营，成功举办中国国际农产品交易会、中国国际体育用品博览会等大型展会，鼓楼区跻身全国楼宇经济十大潜力城区。福州航空新开通16条航线，长乐国际机场年旅客吞吐量突破1000万人次，福州市港口年货物吞吐量1.13亿吨。鼓岭生态旅游区、永泰云顶旅游区获评国家生态旅游示范区。大力推进大众创业、万众创新，新增省级以上创新型企业25家和市级以上企业技术中心20家、院士（专家）工作站16家、众创空间14家，高新技术产业增加值突破1000亿元。

　　加快建设中国福州海西引智试验区，新引进高层次人才34人、人才团队2个。

　　（三）创新体制机制，重点领域改革向纵深推进。稳妥开展经营性国有资产集中统一监管。积极推进政府和社会资本合作，引入民间资金157亿元。完成第三轮简政放权，市级审批服务事项取消49项、下放51项，33项审批服务事项在全国首创“市区同权、多点办理”。建立行政权力清单、责任清单制度，梳理公布市本级7944项行政权力和10013项责任事项。市行政服务中心通过国家级服务业标准化试点单位验收，建成启用全省首个市民服务中心。全面推广“一照一码”登记制度改革，全市新登记企业数增长35.8%。深化户籍制度改革，建立积分落户制度。闽侯国家级农村集体资产股份权能改革试点启动实施，农村土地承包经营权确权登记颁证试点有序推进。

　　（四）拓展发展空间，大开放战略深入实施。福州新区获批成为全国第14个国家级新区，对接新区产业项目200多项、总投资超2500亿元，新区固定资产投资超2000亿元。大力推进自贸区福州片区建设，推出6批54项体制创新举措，其中14项属于全国首创，区内新增企业4535户，注册资本830.13亿元。加快建设21世纪海上丝绸之路核心区，成功举办海丝博览会、亚洲合作对话工商大会、丝路国际电影节、海丝国际旅游节等重大活动，中国—东盟海产品交易所等项目建设有效推进。注重“引进来”、“走出去”并举，新批千万美元以上外（台）资项目74项、境外投资项目73项。深化榕台交流合作，开通黄岐至马祖客运航线，在全国率先启用电子台胞证、成立首家台胞权益保障中心，海峡青年交流营地启动建设。加强榕港榕澳各领域交流合作，在港澳举办推介会成果丰硕。外事、侨务等工作取得新成效，与丹麦霍尔拜克市建立友好交流城市关系。福莆宁岚同城化步伐加快，平潭及闽江口水资源配置、长平高速公路、福平铁路等项目扎实推进。对口支援和山海协作工作有效开展。

　　（五）夯实农业基础，“三农”工作力度不断加大。大力发展都市现代农业，全市农林牧渔业总产值764.87亿元，增长4%。新成立322家农民专业合作社，建成5个省级农业标准化示范区，新建设施大棚3200亩。加快发展远洋渔业，在几内亚比绍等国家新建远洋渔业基地。进一步完善农村基础设施配套，54个水利项目完成投资24.96亿元，建成农村公路244公里，新增更新农村客车135辆，解决农村10.3万人饮水安全问题。江阴、青口省级“小城市”培育试点进展顺利，试点小城镇和美丽乡村建设扎实推进。积极开展新农村“幸福家园工程”建设，完成23个市级示范村185个项目建设。实施“造福工程”搬迁5350人，帮扶2.1万人脱贫。

　　（六）改善宜居品质，城市功能品位持续提升。《福州市城市总体规划（2011—2020）》获得国务院批复，中心城区“多规合一”编制工作基本完成。不断拓展对外通道，合福高铁福州段、沈海复线宁连高速福州段、京台线建闽高速福州段等项目建成通车。加快完善城市路网，新改扩建城市道路71公里，完成城区路网13个节点改造。轨道交通1号线南段试通车，2号线建设加快推进。新增更新公交车1019辆，新辟优化公交线路210条，新增预约出租车113辆，新增公共停车泊位1767个。认真实施“四绿”工程，完成造林绿化13.22万亩，建成开放飞凤山奥体公园、金鸡山栈道等10个城市休闲空间，新建40公里绿道，新增391万平方米城市公共绿地。基本完成飞凤河、台屿河等7条内河整治，实现左海与西湖相通。综合整治123个老旧住宅小区，启动实施旧屋区改造1062万平方米。提高城市防灾减灾能力，江北城区山洪防治及生态补水工程等项目启动建设。道路交通安全、“两违”、非法采砂等专项整治扎实开展，成立“智慧福州”管理服务中心，福州市数字城管系统一期通过国家验收。成功举办中国生态文明论坛福州年会，顺利通过国家生态市建设技术评估和省级森林城市检查验收，全市空气质量位居全国74个重点城市第6位。

　　（七）完善公共服务，各项社会事业协调发展。新增中小学学位5550个、公办幼儿园学位2160个，在五城区试点政府购买普惠性民办、集体办学前教育服务。全面开展公立医院综合改革，市属、区属公立医院实行药品、耗材零差率销售，新建92个村卫生所、30家基层医疗卫生机构“中医馆”。全面启动国家公共文化服务体系示范区创建，第二期26个城市街区24小时自助图书馆建成投入使用，市老年人活动中心、温泉博物馆等项目建成开放，“文化惠民·六进”活动被文化部评为基层文化志愿服务示范项目。加强历史文化遗产有效保护与合理开发，三坊七巷获评亚太地区“文化遗产保护奖”和国家5A级景区。圆满举办第一届全国青运会，赢得了海内外高度评价，福州代表团获得金牌数、奖牌数、总分数在55个城市代表团中均排名第3位。成功举办环福州·永泰国际公路自行车赛、福州国际马拉松赛、中国羽毛球公开赛、全球华人篮球邀请赛等大型赛事。深化双拥共建，积极为部队办实事解难事，军政军民团结进一步巩固。

　　（八）注重民生保障，人民群众幸福感有效增强。全市各级财政用于民生支出565.7亿元，占一般公共预算支出的78.2%，年初确定的市级25项78件为民办实事项目件件有落实。城镇新增就业14.9万人，转移农业富余劳动力4.9万人。覆盖城乡的社会保险体系全面建立，企业退休人员养老金、城乡居民基础养老金、城乡低保、农村“五保”标准和新农合人均筹资水平进一步提高。基本建成保障性安居工程2.5万套，解决8667户群众逾期安置问题。村（居）委会换届选举工作全面完成。安全生产形势保持总体稳定。人民群众安全感达92.86%。

　　（九）践行“三严三实”，政府工作作风切实改进。深入开展“三严三实”专题教育和作风建设年等活动，认真贯彻中央八项规定精神，全市政府系统“三公”经费支出下降15.1%。深入开展效能建设、绩效管理工作，坚决整肃“为官不为”、“庸懒散拖”等问题。行政监察、审计监督等力度持续加大，反腐倡廉工作得到加强。认真执行市人大及其常委会决定决议，自觉接受人大及其常委会的法律监督、工作监督和政协的民主监督，积极支持政协开展协商民主活动。提请市人大常委会审议地方性法规草案3件，制定政府规章和规范性文件29件。办复509件省、市人大代表建议和442件省、市政协提案，满意率分别为97.3%和99.8%。2015年各项工作的完成，标志着“十二五”规划主要目标的胜利实现。过去的五年，是我市经济总量持续增长、综合实力持续提升、人民群众获得感持续增强的五年。尤其是中央作出支持福建加快发展的重大决策部署，批准设立福州新区、自贸区福州片区，支持福建建设21世纪海上丝绸之路核心区和生态文明先行示范区，福州发展迎来了千载难逢的历史性机遇，经济社会发展迈上了一个新的台阶。

　　一是综合实力显著增强。全市生产总值突破5000亿元，人均GDP超过1万美元，一般公共预算总收入、地方一般公共预算收入、固定资产投资等实现比2010年翻一番。福州被评为全国文明城市，并蝉联全国科技进步先进市、全国双拥模范城、国家卫生城市等称号。

　　二是产业结构持续优化。三次产业结构由2010年的9.0∶44.9∶46.1调整为2015年的7.7∶44.2∶48.1。产业不断发展壮大，形成5个千亿产业集群和10家百亿企业（集团）。现代物流、金融、信息服务、商贸、旅游、文化创意、服务外包等服务业加快发展。高新技术产业增加值占GDP比重从13.8%提高到19%。

　　三是城乡面貌明显改观。“东进南下、沿江向海”发展战略加快实施，常住人口城镇化率从62%提高到67.7%。高速公路总里程从396公里增加到587公里，实现县县通高速。城市道路总长从1050公里增加到1256公里，城市轨道交通加快建设，新增污水日处理能力47万吨，启动海绵城市建设，城市保障服务和防灾减灾能力切实增强。建成海峡奥体中心等一批地标性建筑，朱紫坊、上下杭、冶山、烟台山等历史文化街区（风貌区）保护修复工作成效显著。环境综合整治力度不断加大，旧屋区改造、内河整治、“两违”治理、市政提升等工作扎实推进。新农村建设步伐加快，试点小城镇和新农村“幸福家园工程”、美丽乡村建设取得较好成效。节能减排任务全面完成，空气质量、森林覆盖率持续位居全国前列。

　　四是改革开放不断深化。国资监管和国企改革稳步推进，构建形成以四家投资集团与两个专业公司为构架的国资监管格局。民营经济持续健康发展，“回归工程”成效显著。政府机构改革和投融资、财税、科技、教育、文化、卫生、户籍等领域改革扎实推进。农村综合改革向纵深拓展，土地股份合作经营、林权流转等试点工作有效开展。对外开放水平进一步提高，实际利用外商直接投资累计达73.76亿美元，来榕投资或设立机构的世界500强企业增加到93家。榕台交流合作先行先试取得新进展，在榕台资银行实现零的突破，福州被列为赴台个人游试点城市。福莆宁岚同城化步伐加快，榕港榕澳合作、海内外友城合作、闽浙赣皖经济协作、闽东北区域协作、山海协作、对口支援以及外事、侨务、异地商会等工作富有成效。

　　五是社会事业有效提升。各级各类教育协调发展，率先在省内实现全市县域义务教育基本均衡，在线德育、体艺美育、幸福教育等工作走在全省前列。覆盖城乡的医疗卫生服务体系、疾病预防控制体系和突发公共卫生事件应急体系进一步完善，基本医疗和公共卫生服务覆盖所有行政村，在全省率先组建多种形式医疗联合体。公共文化服务不断拓展，“城市15分钟、农村30分钟公共文化服务圈”初步形成。成功举办第一届全国青运会、央视中秋晚会等重大文体活动。三坊七巷、“海上丝绸之路：福州史迹”入选中国世界文化遗产预备名单，“福州茉莉花与茶文化系统”入选全球重要农业文化遗产。人口计生、民族宗教、民政、新闻出版、广播影视、哲学社会科学、科普、防震、气象、地方志、档案、老龄、妇女儿童、残疾人等事业全面发展。

　　六是人民生活切实改善。全市各级财政累计用于民生支出1935.25亿元，累计为城乡人民兴办实事项目115项431件。城镇居民人均可支配收入、农村居民人均可支配收入分别比2010年增长67.5%、82.3%。累计实现城镇新增就业73.95万人、转移农业富余劳动力26.7万人。城乡居民基本养老保险制度实现全覆盖，城乡低保、农村“五保”和城乡居民医保财政补助标准持续提高。累计建成保障性安居工程11.6万套，实施旧屋区改造2590万平方米，解决1.6万户低收入家庭住房困难。社会救助、社会福利、社会优抚、社会慈善加快发展，各类扶贫济困活动深入开展。“平安福州”建设扎实推进，信访、行政复议、人民调解、普法、法律援助、法律服务、社区矫正、青少年事务社工以及国防动员、双拥共建、民兵预备役建设、海防、人防、反走私等工作进一步加强。

　　各位代表，成绩来之不易，这是在省委、省政府和市委的正确领导下，全市人民团结奋斗，社会各界大力支持的结果。在此，我代表市人民政府，向全市人民致以崇高的敬意！向人大代表、政协委员、各民主党派、工商联、各人民团体、无党派人士、离退休老同志和社会各界人士，向中直、省直机关企事业单位和驻榕部队、武警官兵、公安政法干警，向关心支持福州发展的港澳台同胞、海外侨胞和国际友人，表示衷心的感谢！

　　在看到成绩的同时，我们也必须正视当前福州发展中存在的困难和问题，主要是：经济下行压力仍然较大，部分行业、企业生产经营困难，稳增长任务较为艰巨；产业结构不够优、竞争力不够强，自主创新能力不足，转型升级任重道远；城乡规划建设管理水平有待进一步提升，交通拥堵、违法建设等问题还比较突出，防灾减灾、防洪排涝、停车场所、地下管网等设施还比较薄弱；城乡之间、县（市）区之间发展还不平衡；节能减排压力较大，环境资源约束与经济社会发展之间矛盾比较突出；住房、教育、医疗、物价、食品安全、社会保障等与群众的要求还有较大差距；社会管理还需加强，安全生产事故、群体性事件仍时有发生；一些公务人员仍然存在“为官不为”、“庸懒散拖”等问题。我们要切实采取有效措施，认真加以解决。

　　二、“十三五”发展暨2016年工作安排

　　各位代表，根据《中共福州市委关于制定福州市国民经济和社会发展第十三个五年规划的建议》编制的《福州市国民经济和社会发展第十三个五年规划纲要（草案）》，提出了今后五年经济社会发展的指导思想、目标任务和政策措施，地区生产总值提前实现比2010年翻一番，到2020年一般公共预算总收入超过1250亿元、地方一般公共预算收入超过780亿元。

　　“十三五”时期我市国民经济和社会发展的指导思想是：高举中国特色社会主义伟大旗帜，全面贯彻党的十八大和十八届三中、四中、五中全会精神，深入贯彻习近平总书记系列重要讲话精神和对福建、福州工作的重要指示，坚持“四个全面”战略布局，坚持发展第一要务，树立创新、协调、绿色、开放、共享的发展理念，以跨越赶超为主题，以福州新区为引领，以转型升级为主线，以改革开放为动力，以提升人民群众获得感为目的，统筹推进经济建设、政治建设、文化建设、社会建设、生态文明建设和党的建设，率先全面建成小康社会，努力建设更具实力、更富活力、更有魅力的现代化新福州。

　　我市“十三五”发展的主要任务：一是推进产业转型升级。优先发展现代服务业，大力发展先进制造业，培育发展战略性新兴产业，规模以上工业增加值达到3000亿元以上，力争服务业增加值突破5000亿元，培育形成4个产值超2000亿元、2个产值超1000亿元的产业集群以及20家百亿企业（集团），加快打造东南沿海先进制造业重要基地、现代金融中心、文化创意之都、海峡会展之都、总部经济高地、海洋经济强市以及全国重要旅游目的地。二是突出创新驱动引领。大力推进自主创新，建立健全以企业为主体协同创新的技术创新体系，实现省级以上企业工程技术研究中心突破100家，市级以上企业技术中心达250家，建设中国福州海西引智试验区，创建国家级自主创新示范区。三是提高改革开放水平。全面推进服务型政府建设和资源要素市场化改革，进一步激发市场主体活力。积极融入国家“一带一路”战略，全力推进福州新区开放开发，加快建设自贸区福州片区和21世纪海上丝绸之路核心区。深化两岸经贸合作，推动两岸人文融合，打造对台往来综合枢纽。四是推进福州大都市区建设。全面实施新一轮城市总体规划，加快中心城市“东进南下、沿江向海”发展步伐，进一步凸显滨江滨海现代化国际大都市格局。努力完善对内对外综合交通体系、城市公共服务体系、现代管理体系、历史文化体系、生态环境体系和安全保障体系，不断提升城市内涵品质。充分发挥省会中心城市的龙头带动作用，推动福莆宁岚连片繁荣、共同发展。五是统筹城乡一体发展。加快推进新型城镇化建设，实现常住人口城镇化率达75%左右，全面形成以福州新区为引领、中心城区为龙头、中小城市和小城镇协调发展、新型城镇化和新农村建设协调推进的格局。加大科学扶贫、精准扶贫力度，深化县（市）区对口协作，力争2017年底实现扶贫对象全部脱贫。六是构建文明和谐生态。深入实施生态文明战略，持续提升国家生态市建设水平，建成国家森林城市。大力发展绿色经济，推广以园区为依托的循环经济模式，加大生态修复和环境保护力度，健全生态文明制度体系，积极建设生态文明先行示范区。七是切实保障改善民生。努力提高居民收入水平，加快推进基本公共服务均等化，构建完善公共就业创业服务体系、现代教育体系、公共卫生体系、社会保障体系、现代公共文化服务体系和公共安全体系。

　　各位代表，2016年是全面建成小康社会决胜阶段的开局之年，也是推进结构性改革的攻坚之年。我们要进一步坚定信心，牢固树立和贯彻落实五大发展理念，积极适应经济发展新常态，坚持稳中求进工作总基调，统筹稳增长、调结构、强动力、惠民生、防风险，加强供给侧结构性改革，着力去产能、去库存、去杠杆、降成本、补短板，确保“十三五”发展开好局起好步。今年经济社会发展的主要预期目标是：地区生产总值增长9.5%，力争更快更好些；地方一般公共预算收入增长7.5%；固定资产投资增长12%；外贸出口增长3%；实际利用外商直接投资增长7%；社会消费品零售总额增长13%；居民消费价格总水平涨幅控制在3%左右；城镇居民人均可支配收入增长8.5%左右，农村居民人均可支配收入增长9%；城镇登记失业率控制在3.5%以内；确保完成省下达的节能减排降碳任务。　为实现上述预期目标，重点抓好以下七个方面工作：

　　（一）全面加快福州新区开放开发

　　突出规划引领和机制创新。围绕“三区一门户一基地”的战略定位，认真开展“新区建设年”各项工作，抓紧推进《福州新区发展规划》、《福州新区总体规划（2015—2030）》的编制和相关报批工作。进一步健全完善新区规划体系，启动新区范围内基础设施规划、公共服务设施布局规划以及相关专项规划、重点区域控制性详规、新区城市设计研究等工作。加快完善新区行政管理体制和运行机制，努力实现经济管理扁平化、社会管理精细化、政府服务高效化。组建新区开发投资集团，设立若干投资平台，协调推进各片区开发建设。推进福州新区与平潭一体化联动发展，加快形成政策共用、利益分享的互利共赢合作机制。

　　加快基础设施建设。推进滨海大通道、东部快速通道二期、道庆洲大桥、马尾大桥、环南台岛滨江休闲路、江涵大桥等一批重大交通设施及新区供电供水排水、生态环卫等基础设施体系建设，策划一批教育、医疗、消费等城市配套设施项目。扎实推动三江口、闽江口、滨海新城、福清湾、江阴湾等五大城市功能组团建设，启动海峡中央商务区建设，积极打造临空经济区、闽台（福州）蓝色经济产业园、江阴工业集中区等一批重大园区。初步安排新区重点项目391项，年度计划投资约1600亿元，争取新区完成固定资产投资3000亿元。

　　强化产业支撑。推进新区产业布局调整，抓好中铝铝精深加工、三峡集团海上风电装备产业园、中核建核电产业园、中车株机集团新能源等一批项目落地动建，加快推进京东方8.5代面板、聚酰胺一体化、恒申合纤、中景石化科技园、福清核电3号~6号机组等一批项目建设，抓紧引进一批符合新区产业发展方向的高新技术产业、战略性新兴产业项目，推动新区建设与产业升级“双轮驱动”、协同发展，着力打造东南沿海重要的现代产业基地，确保新区GDP增长速度高于全市2个百分点。

　　（二）持续深化改革扩大开放

　　加快重点领域改革。深化国有企业改革，全面推进市属投资集团建设，加快国有企业负责人薪酬制度改革。进一步创新投融资方式，鼓励发展投资基金。大力发展民营经济，支持民营资本以多种方式进入基础产业、社会事业以及特许经营领域。清理规范中介服务和前置审批，全面实施清单管理，推行企业电子执照和全程电子化登记。继续加大政府购买服务力度，推进道路保洁、垃圾收运和市政设施管养、内河整治管养等领域的市场化运作。全面开展不动产统一登记。加快推进农村集体建设用地和宅基地使用权确权登记颁证。深化财政体制改革，健全政府预算体系。继续完善市行政服务中心、市民服务中心，整合建立统一的公共资源交易服务中心。

　　推进自贸试验区建设。推出一批可复制推广的创新举措，加快创新成果的复制推广，推动试验区内外联动发展。建立“一站式”办结审批机制，加强事中事后监管。引导进出口企业在福州片区注册，加快江阴整车进口口岸、跨境电商产业园、两岸金融创新合作示范区、海丝商城、利嘉保税商品展示交易中心、海峡智贸城等平台建设，做大福州片区规模。积极推进两岸金融合作和人民币跨境使用、外汇管理等领域改革。大力推动海关特殊监管区整合优化。

　　打造海丝战略枢纽城市。加快长乐机场二期、福厦客专、绕城高速东南段、莆炎高速福州段、长福高速公路、牛头湾作业区12号、13号泊位等一批项目建设，争取开通更多的海港、空港国际航线，建设“一带一路”通陆达海的重要枢纽。支持中国—东盟海产品交易所建设海外分中心，加快境外养殖基地建设，力争境外养殖投产面积达到1.5万亩。不断扩大海丝博览会、丝路国际电影节、海丝国际旅游节等活动影响力，拓宽与“海丝”沿线国家、地区合作的范围和领域，推动“海上丝绸之路：福州史迹”申报世界文化遗产，建成开放福州海上丝绸之路展示馆。密切与侨团、商会的联系，做好华裔新生代工作。加大精准招商力度，强化与央企、世界500强企业和国际知名公司合作。积极开拓出口市场，大力发展服务贸易和技术贸易。支持企业加大先进技术引进和关键装备进口力度。鼓励企业“走出去”发展，培养本土跨国公司。加快推进电子口岸建设，争取罗源湾港区扩大开放通过国家级验收。

　　拓展对台等交流合作。加快台商投资区、农业合作试验区、台湾农民创业园等平台建设。支持榕台合资设立产业投资基金、创业投资基金，争取建立国家对台金融合作示范区。深化榕台科技、教育、文化、卫生、体育以及宗教民俗、祖地文化等领域的全方位交流，继续办好“5·18”海交会、海峡青年节、渔业周·渔博会、海峡两岸合唱节等一批对台特色交流活动，推进台湾青年创业基地建设，完成海峡青年交流营地一期建设。大力发展海峡旅游，加快构建环马祖澳旅游区。进一步深化与港澳在金融、物流、旅游、文化创意等领域的务实合作。加强泛珠三角、闽浙赣皖、闽东北的区域协作、山海协作，继续抓好对口援疆援藏援宁工作。

　　推动福莆宁岚互动联动发展。主动争取平潭综合实验区政策辐射，促进平潭与周边地区有机联动发展。支持平潭建设国际旅游岛，加快建设平潭及闽江口水资源配置、长平高速公路、福平铁路等项目。继续在科技、教育、卫生、社保、人才培训等方面为平潭提供良好服务。加快福莆宁岚在交通建设、产业发展、公共服务等领域的同城化步伐，促进福莆宁岚协同发展。

　　（三）着力打造产业升级版

　　推动主导产业高端化集聚化发展。大力实施“中国制造2025”，抓好一批具有产业带动力的重大项目建设，完善产业链关键环节和上下游配套，做大做强产业集群。推进新一轮企业技改，完成技改投资830亿元以上，抓好130项重点技改项目。积极引导企业应用数控技术和智能装备进行智能化技术改造，推动纺织服装、电子通信器件、汽车零部件、食品、金属制品等行业开展“机器换工”。实施商标、品牌和质量强市战略，支持企业收购国际知名品牌，加快培育一批驰名商标、名牌产品。

　　促进新产业、新业态、新模式蓬勃发展。推进通用航空产业园、普天集团新能源汽车动力电池、大唐新能源闽侯青林风力发电、神华煤港电一体化、华能罗源港电储基地、永泰抽水蓄能电站等项目建设，推动新一代信息技术、生物与新医药、新材料、新能源、节能环保、高端装备制造等新兴产业规模化发展。大力推广新能源汽车，加快充电桩（站）建设。积极推进“互联网+”，依托国家新型工业化产业示范基地（物联网）、福州高新区、数字福建（长乐）产业园、中国福州（仓山）互联网+产业园等平台，加快发展大数据、物联网、云计算等产业，推动互联网与制造业、农业等深度融合、协同发展。努力创建“中国软件名城”，促进中海创、新大陆、网龙、星网锐捷、瑞芯微等企业做大做强。

　　推进现代服务业质量提升、比重提高。抓好鼓楼区国家服务业综合改革试点，发展壮大海峡金融商务区、闽江北岸中央商务区、国际创新中心贵安基地等总部经济集聚区。加快国家电子商务示范城市建设，发挥海峡两岸电子商务经济合作实验区、海峡电子商务产业基地带动效应，积极引进跨境电商龙头企业。持续推进大东街口商圈改造提升，加快华润万象城等城市综合体建设。优化完善物流规划布局和城市配送体系，大力发展第三方物流、冷链物流、智慧物流、电子商务物流，推动闽台电子商务与现代物流园等项目建设。积极创建服务外包示范城市。加快建设海西现代金融中心，支持发展民营银行、小额贷款公司、股权投资机构和融资租赁公司。规范民间融资行为，有效防范和化解金融风险。稳定住房消费，打通保障房与商品房转换通道，发展住房租赁市场，着力化解房地产库存。完善智慧旅游体系，突出发展清新生态、闽都文化、温泉休闲、海峡、海丝和乡村等旅游品牌。办好中国国际体育用品博览会、中国糖酒商品交易会等大型展会。

　　提升都市现代农业发展水平。实施“粮安工程”，确保粮食播种面积稳定在160万亩以上。做大特色优势农业和品牌农业，加快金鱼产业园等一批特色农业项目建设。发挥“一区两园”农民创业示范基地作用，加强农田水利基础设施建设，新增设施大棚1000亩，扶持闽清等省定农产品主产地建设。提升农业设施化和智能化水平，积极发展循环农业、休闲观光农业和林下经济。大力发展远洋捕捞和海产品精深加工，推进海峡现代渔业经济区、马尾东盟海洋产业园等项目建设。鼓励发展绿色高效生态养殖，力争完成罗源湾养殖退养。引导和规范土地经营权有序流转，加快培育一批专业大户、农民专业合作社、家庭农场等新型农业经营主体，积极培养新型职业农民。加大良种繁育和农业科技应用推广力度。完善农业社会化服务体系，促进农业标准化生产。

　　实施创新驱动战略。支持以企业为主承担重大科技专项等创新项目，开展关键技术研发攻关和协同创新，努力创建国家级自主创新示范区。加快建设中科院海西研究院、福州技术市场等创新示范基地，进一步密切产学研用对接合作。全力推动大众创业、万众创新，加快大学生创业园建设，筹建电商创业孵化基地，新建科技孵化器5家、众创空间10家，争创国家小微企业创业创新基地城市。发挥中国福州海西引智试验区作用，加强与国内外知名高校、科研院所、榕籍院士的合作，积极培养和引进创新创业各类人才。

　　强化企业帮扶。加大中小微企业、民营企业扶持力度，积极帮助企业降低制度性交易成本、税费负担、社会保险费、财务成本、电力价格和物流成本。积极化解产能过剩，鼓励企业通过技改、兼并重组、上市等方式做大做强，尽可能多兼并重组、少破产清算。支持企业开拓市场，提高名特优新产品市场占有率。加强政银企合作，充分发挥各级企业应急保障资（四）统筹推进城乡一体化发展

　　提升城市规划建设水平。完成全市“多规合一”工作，全面开展城市设计，增强城市规划的科学性和权威性。推进基础设施建设，新建改造污水管网、供水管网、雨水管网各150公里以上，新建地下综合管廊22.9公里。积极构建快速交通网络，加快福州外郊快速环线、城区北向第二通道、福马路提升改造工程等项目建设，建成湖东东路下穿、南台大道等市政项目。进一步打通断头路，优化社区交通微循环。加快轨道交通建设，实现1号线全线贯通，全面建设2号线、6号线，力争启动4号线、5号线建设。加强地下空间开发利用，推进万宝商圈地下人防工程等项目建设。鼓励发展立体停车、地下停车，新增公共停车泊位6000个，建成一批立体停车库。深入开展“公交都市”创建活动，新增更新公交车350辆，新辟优化公交线路20条，继续推广公共便民自行车。切实提高城市防灾减灾能力，积极申报第二批国家海绵城市建设试点，推进江北城区山洪防治及生态补水工程、闽江下游南港防洪、霍口水库和东湖、南湖等项目建设，做好城市防洪排涝、防抗台风海浪以及山区地质灾害防范等工作。加快推进区域重点输变电项目建设，优化电力设施布局。

　　加强城市精细化管理。不断完善城市管理体制，全面拓展“智慧城市”管理服务平台应用，加强“数字城管”建设，争创新型智慧城市标杆市。完成流花溪、梅峰河等7条内河整治，启动胪雷河、螺洲河等6条内河整治，开展黑臭水体专项治理。实施旧屋区改造380万平方米，综合整治老旧住宅小区60个、背街小巷36条。新改扩建金山公园、南公园、烟台山公园等10个公园，新建完善城市绿道和慢行系统30公里，造林绿化8.2万亩。加强交通环境治理，科学组织重要节点交通，健全市域交通导识标志。全面落实“门前三包”责任制，有序推进垃圾分类工作。持续治理“两违”、非法采砂、渣土车违规等突出问题。

　　推进新型城镇化和美丽乡村建设。加强中心城区以及县（市）城关周边小城镇的统筹规划和功能配套，编制完成新型城镇化规划。深化户籍制度改革，全面实施居住证制度，实施差别化落户政策，引导和鼓励人口优先向城镇转移。加快江阴、青口省级“小城市”培育试点步伐，吸引社会资本参与小城镇建设，形成一批功能完善的特色小城镇。深入实施新农村“幸福家园工程”，积极打造美丽乡村，争取建成130个美丽乡村、12个美丽乡村精品示范村，加强历史文化名镇名村、传统村落保护工作。关爱农村“三留守”群体，进一步提高农村基本公共服务保障能力。

　　（五）巩固提高生态文明建设水平

　　加强生态系统建设。完成国家生态市考核验收。建立生态保护红线制度，实施生态环境分区分级管控。完善生态补偿和资源有偿使用制度，推进水权、排污权有偿使用和交易试点，开展森林生态效益补偿和重点生态区位商品林赎买工作。健全环境监测网络，严肃生态环保责任追究，严厉打击环境违法行为。提高全民环保意识，大力倡导和推广节约资源能源、低碳出行和绿色消费。加强闽江河口湿地等重要湿地以及自然保护区、沿海防护林等重点生态功能区的保护，积极创建国家级海洋生态文明示范区。

　　推进节能减排。全面实施能耗强度、碳排放强度和能源消费总量控制，落实重点行业脱硫脱硝工作，继续实行县（市）区污染物减排刚性约束。严格落实环境准入制度，健全落后产能退出机制，坚持绿色发展、循环发展、低碳发展，加快循环经济示范城市建设。实施30项节能技术改造项目、60项重点减排项目，加快建陶等行业清洁能源改造。推进建筑产业化和绿色建筑、建筑节能，大力推广装配式建筑。继续加快环保基础设施建设，启动建设餐厨垃圾处理厂、闽侯生活垃圾处理厂、危险废物处置新场，推进红庙岭垃圾焚烧发电厂三期、连坂污水处理厂二期及厂外管网、闽侯窗厦渣土资源综合利用等项目建设，全面完成城镇污水处理厂污泥的规范化处理处置。

　　深化环境综合治理。加强大气污染防治，全面推进施工扬尘、垃圾焚烧、餐饮油烟污染等十项大气污染整治专项行动，保持空气质量位居全国74个重点城市前列，努力实现“保十争五”的目标。严格落实“河长”责任制，抓好畜禽养殖、石板材等重点行业污染治理和饮用水源地保护。强化对重金属、危险废物、危险化学品、持久性有机污染物、核设施和放射源安全监管，建立完善突发环境事件预防和预警体系。加大水土流失、工矿废弃地恢复治理力度。

　　（六）协调发展各项社会事业

　　推进优质教育均衡化。实施第二期学前教育三年行动计划，新增中小学学位6000个、公办幼儿园学位2220个。推动普通高中多样化发展。加大教育资源配置向农村特别是边远、贫困、民族乡村倾斜力度，全面推进农村义务教育薄弱学校“委托管理”。大力开展素质教育，加快建设青少年校外活动中心。加强产教融合、校企合作，建成市职业教育公共实训基地二期。提高高等教育质量，完善大学城管理体制。强化教师队伍师德教育和专业培养，实施教育高端人才5年培养规划和乡村教师支持计划。支持民办教育发展，鼓励中外合作办学，加快中加国际学校等项目建设。抓好社区教育、特殊教育、老年教育、继续教育、终身教育。

　　打造“健康福州”。深化公立医院综合改革，鼓励和规范社会力量举办医疗机构。持续优化医疗资源布局，加快建设市一医院、市妇幼保健院等重点项目。提高基层医疗服务能力，加强全科医生和乡村医生队伍建设。完善分级诊疗制度，鼓励医师多点执业。强化医德医风建设，构建和谐医患关系。加强卫生应急队伍装备建设，提升疾病预防控制和突发公共卫生事件应急处置能力。支持中医药事业和中西医结合发展。持续开展爱国卫生运动。全面实施两孩政策，促进人口均衡发展。推动体育事业和体育产业发展，继续办好环福州·永泰国际公路自行车赛、福州国际马拉松赛、中国羽毛球公开赛、国际沙滩排球巡回赛、中华龙舟大赛等一批大型赛事，广泛开展全民健身活动，打造“10分钟体育健身圈”。

　　提升城市文化软实力。推进国家公共文化服务体系示范区建设，加快海峡文化艺术中心、海峡青少年活动中心等项目建设，建成开放海峡图书馆、海峡妇女儿童活动中心，启动基层公共文化设施达标、提升工程，打造“15分钟公共文化服务圈”。大力挖掘和弘扬闽都文化，加强文化遗产保护，持续推进朱紫坊、上下杭、冶山、烟台山等历史文化街区（风貌区）保护修复与合理利用，强化非物质文化遗产保护传承。大力支持文艺创作，促进哲学社会科学、新闻出版、广播影视、文学艺术等事业繁荣发展。认真编纂福州市志和年鉴。鼓励发展文化产业，加快中国船政文化城、海峡非物质文化遗产生态园、海西动漫创意之都等文化产业园区建设。

　　巩固文明城市建设成果。大力开展以中国梦和社会主义核心价值体系建设为引领的精神文明建设，加强社会公德、职业道德、家庭美德和个人品德教育。推进群众性精神文明创建活动，深化文明城市、文明村镇、文明单位（行业）、文明家庭、文明校园创建。强化未成年人思想道德建设，鼓励发展志愿服务，培育良好的社会风尚。推进诚信制度化建设，着力构建“诚信福州”。

　　（七）不断提升人民群众获得感

　　打好脱贫攻坚战。重点抓好闽清、永泰等扶贫开发重点县和老少边贫岛地区发展，全年实现脱贫2.07万人，完成“造福工程”搬迁4000人。坚持精准识别，规范建档立卡，摸实摸清底数，确保扶贫对象到户到人。坚持精准施策，分类制定帮扶措施，实行发展产业脱贫、转移就业脱贫、“造福工程”搬迁脱贫、发展教育脱贫、生态补偿脱贫、低保兜底脱贫、医疗保险和医疗救助脱贫。坚持精准管理，建立贫困户脱贫退出认定机制，做到贫困人员应进则进、应退则退。

　　做好就业和社会保障工作。完善就业创业扶持体系，促进结构性失业人员、高校毕业生、农村转移劳动力、就业困难人员和退役军人就业，争取城镇新增就业13.3万人。推行企业工资集体协商，逐步提高最低工资标准，构建和谐劳动关系。进一步提高城乡居民基础养老金、城乡低保、农村“五保”和城乡居民医保财政补助标准，执行全市统一的城乡医保一体化政策，完善对被征地、收海农民的多元保障机制。加快保障性安居工程建设，新开工建设1.8万套，基本建成1万套。积极应对人口老龄化，健全城乡养老服务体系，加强养老服务机构建设与管理，鼓励社会力量参与养老服务，加快养老服务业和健康服务业发展，推进医养结合。保障妇女儿童和青少年合法权益，积极发展社会福利、社会救助、社会慈善和残疾人事业。继续加强“菜篮子”工程建设，新建蔬菜基地3000亩，着力保障市场供应量足、质优、价稳。持续治理“餐桌污染”，建设“食品放心工程”，确保人民群众“舌尖上的安全”。

　　加强和创新社会治理。深入推进“平安福州”建设，进一步完善社会治安防控体系，强化社会治安网格化管理，依法防范和惩治各类违法犯罪。强化安全生产监管和隐患排查治理，坚决防范和遏制重特大事故发生。依法处理信访事项，完善人民调解、行政调解、仲裁调解、司法调解等多元化联动体系，推进矛盾纠纷排查化解。探索建立社区民主协商机制。做好社区矫正工作。健全重大决策社会稳定风险评估机制和突发事件应急处置机制。深入实施“七五”普法。推动社会组织多元健康发展，支持工会、共青团、妇联、残联等人民团体发挥更大作用。加强民族工作，依法管理宗教事务，促进民族团结进步和宗教关系和谐。扎实做好防震减灾、科普、气象、档案、保密等工作。巩固提升双拥模范城创建成果，支持驻榕部队和武警部队建设，加强国防教育、国防动员、国防后备力量建设和海防、人防、反走私等工作，推进军民融合深度发展。

　　三、切实加强新形势下政府自身建设

　　各位代表，新形势新任务对政府自身建设提出了新的更高要求，市人民政府将严守政治纪律和政治规矩，加强和改进政府作风，着力打造法治政府、廉洁政府和服务型政府。

　　坚持依法行政。完善行政机关领导干部及工作人员学法、守法、用法制度，进一步提高依法行政水平。坚持用制度管权管事管人，严格依照法定权限和程序行使权力、履行职责，完善重大事项听证、政府法律顾问等制度，把权力关进制度的笼子里。自觉接受人大及其常委会的法律监督、工作监督和政协的民主监督，支持协商民主广泛多层制度化发展，切实提高办理人大代表建议、政协提案的满意率、落实率。强化权力公开运行，积极推行政务公开、政府信息公开，主动接受舆论和群众的监督。

　　强化担当尽责。深化行政管理体制改革，进一步转变政府职能，持续推进简政放权，切实提高政府服务效能。弘扬敢想、敢闯、敢干、敢担的干事创业精神，把心思和劲头放在苦干实干、争创一流上，勇于直面矛盾、攻坚克难，切实做到想干事、会谋事、敢担事、能成事。大力弘扬“马上就办、真抓实干”的优良传统，坚决纠正“为官不为”、“庸懒散拖”等问题，全面提高政府执行力和公信力。

　　秉持执政为民。巩固拓展“三严三实”专题教育活动成果，深入开展“三比一看”、“四个万家”、“企业服务月”等活动，努力为企业和群众排忧解难。坚持权为民所用，持续整改“不严不实”问题，坚决纠正损害群众利益的不正之风，切实提高群众的满意度。认真执行《中国共产党廉洁自律准则》、《中国共产党纪律处分条例》，严格落实中央八项规定精神，加强行政监察和审计监督，积极推进审计监督全覆盖，营造风清气正的良好氛围。

　　各位代表，建设现代化新福州是全市人民的共同期盼，也是时代赋予我们的崇高使命。让我们紧密团结在以习近平同志为总书记的党中央周围，在省委、省政府和市委的领导下，进一步解放思想、开拓进取，为全力推进福州新区开放开发，在更高起点上加快建设闽江口金三角经济圈，加快建设更具实力、更富活力、更有魅力的现代化新福州而努力奋斗！金和农业产业化龙头企业“互助资金池”的作用，帮助企业解决资金周转困难和融资难问题。
